# Supplementary material for: Transcriptional Reprogramming of Autographa Californica Multiple Nucleopolyhedrovirus Chitinase and Cathepsin Genes Enhances Virulence
Source: Viruses. 2023 Feb 11;15(2):503. doi: 10.3390/v15020503 (PMC9965964; doi:10.3390/v15020503)

## Slide 1
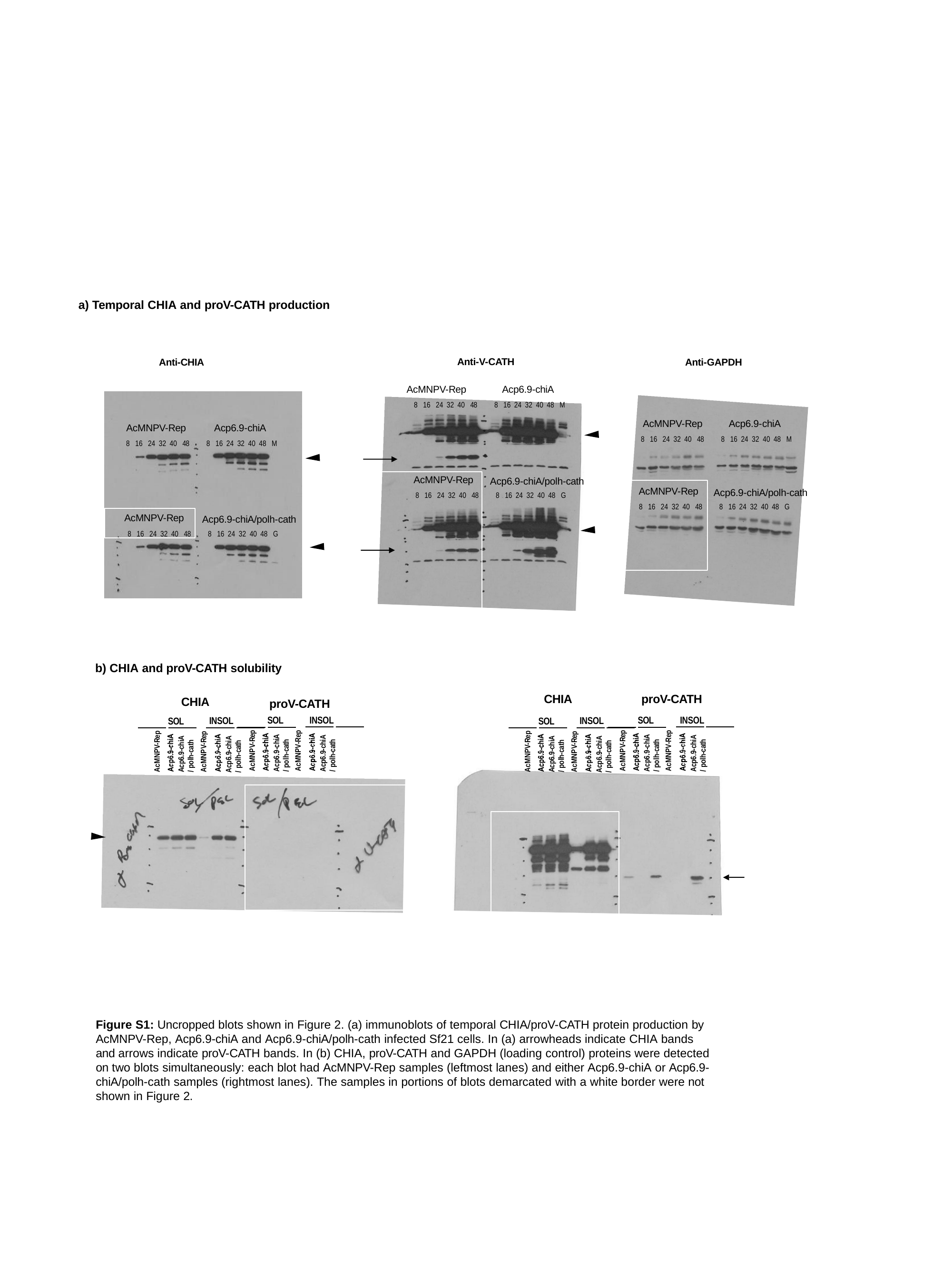

a) Temporal CHIA and proV-CATH production
Anti-V-CATH
Anti-CHIA
Anti-GAPDH
AcMNPV-Rep
8 16 24 32 40 48
Acp6.9-chiA
8 16 24 32 40 48 M
AcMNPV-Rep
8 16 24 32 40 48
Acp6.9-chiA
8 16 24 32 40 48 M
AcMNPV-Rep
8 16 24 32 40 48
Acp6.9-chiA
8 16 24 32 40 48 M
AcMNPV-Rep
8 16 24 32 40 48
Acp6.9-chiA/polh-cath
8 16 24 32 40 48 G
AcMNPV-Rep
8 16 24 32 40 48
Acp6.9-chiA/polh-cath
8 16 24 32 40 48 G
Acp6.9-chiA/polh-cath
8 16 24 32 40 48 G
AcMNPV-Rep
8 16 24 32 40 48
b) CHIA and proV-CATH solubility
CHIA
SOL
proV-CATH
SOL	INSOL
proV-CATH
SOL	INSOL
CHIA
INSOL
INSOL
SOL
Acp6.9-chiA
/ polh-cath
AcMNPV-Rep
Acp6.9-chiA
/ polh-cath
AcMNPV-Rep
Acp6.9-chiA
/ polh-cath
AcMNPV-Rep
Acp6.9-chiA
/ polh-cath
AcMNPV-Rep
AcMNPV-Rep
AcMNPV-Rep
Acp6.9-chiA
/ polh-cath
AcMNPV-Rep
Acp6.9-chiA
/ polh-cath
AcMNPV-Rep
Acp6.9-chiA
/ polh-cath
Acp6.9-chiA
/ polh-cath
Figure S1: Uncropped blots shown in Figure 2. (a) immunoblots of temporal CHIA/proV-CATH protein production by AcMNPV-Rep, Acp6.9-chiA and Acp6.9-chiA/polh-cath infected Sf21 cells. In (a) arrowheads indicate CHIA bands and arrows indicate proV-CATH bands. In (b) CHIA, proV-CATH and GAPDH (loading control) proteins were detected on two blots simultaneously: each blot had AcMNPV-Rep samples (leftmost lanes) and either Acp6.9-chiA or Acp6.9- chiA/polh-cath samples (rightmost lanes). The samples in portions of blots demarcated with a white border were not shown in Figure 2.

## Slide 2
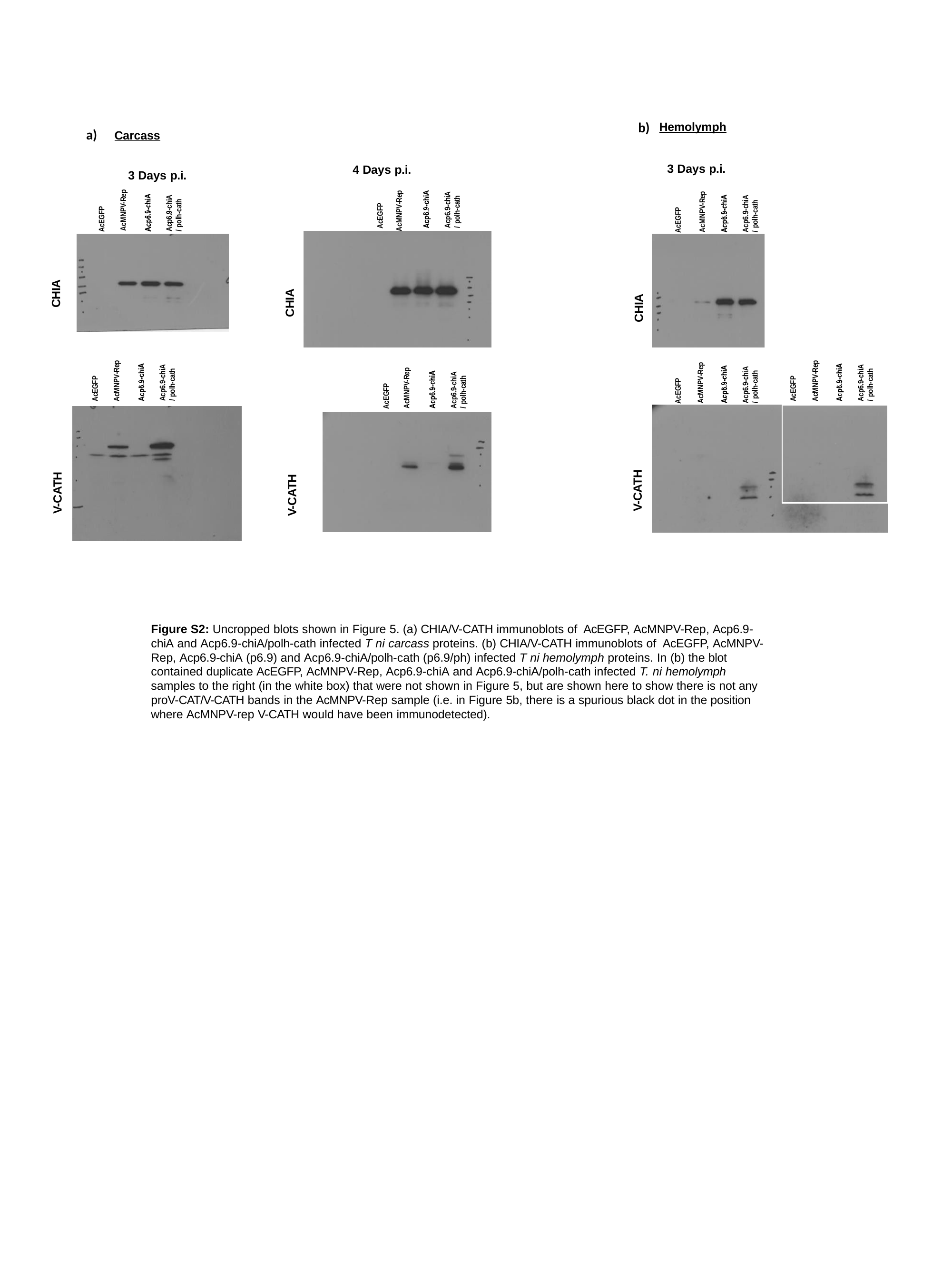

b)
Hemolymph
a)	Carcass
3 Days p.i.
4 Days p.i.
3 Days p.i.
AcMNPV-Rep
AcMNPV-Rep
AcMNPV-Rep
Acp6.9-chiA
/ polh-cath
Acp6.9-chiA
/ polh-cath
Acp6.9-chiA
/ polh-cath
AcEGFP
AcEGFP
AcEGFP
CHIA
CHIA
CHIA
AcMNPV-Rep
AcMNPV-Rep
AcMNPV-Rep
Acp6.9-chiA
/ polh-cath
Acp6.9-chiA
/ polh-cath
Acp6.9-chiA
/ polh-cath
AcMNPV-Rep
Acp6.9-chiA
/ polh-cath
AcEGFP
AcEGFP
AcEGFP
AcEGFP
V-CATH
V-CATH
V-CATH
Figure S2: Uncropped blots shown in Figure 5. (a) CHIA/V-CATH immunoblots of AcEGFP, AcMNPV-Rep, Acp6.9- chiA and Acp6.9-chiA/polh-cath infected T ni carcass proteins. (b) CHIA/V-CATH immunoblots of AcEGFP, AcMNPV- Rep, Acp6.9-chiA (p6.9) and Acp6.9-chiA/polh-cath (p6.9/ph) infected T ni hemolymph proteins. In (b) the blot contained duplicate AcEGFP, AcMNPV-Rep, Acp6.9-chiA and Acp6.9-chiA/polh-cath infected T. ni hemolymph samples to the right (in the white box) that were not shown in Figure 5, but are shown here to show there is not any proV-CAT/V-CATH bands in the AcMNPV-Rep sample (i.e. in Figure 5b, there is a spurious black dot in the position where AcMNPV-rep V-CATH would have been immunodetected).

## Slide 3
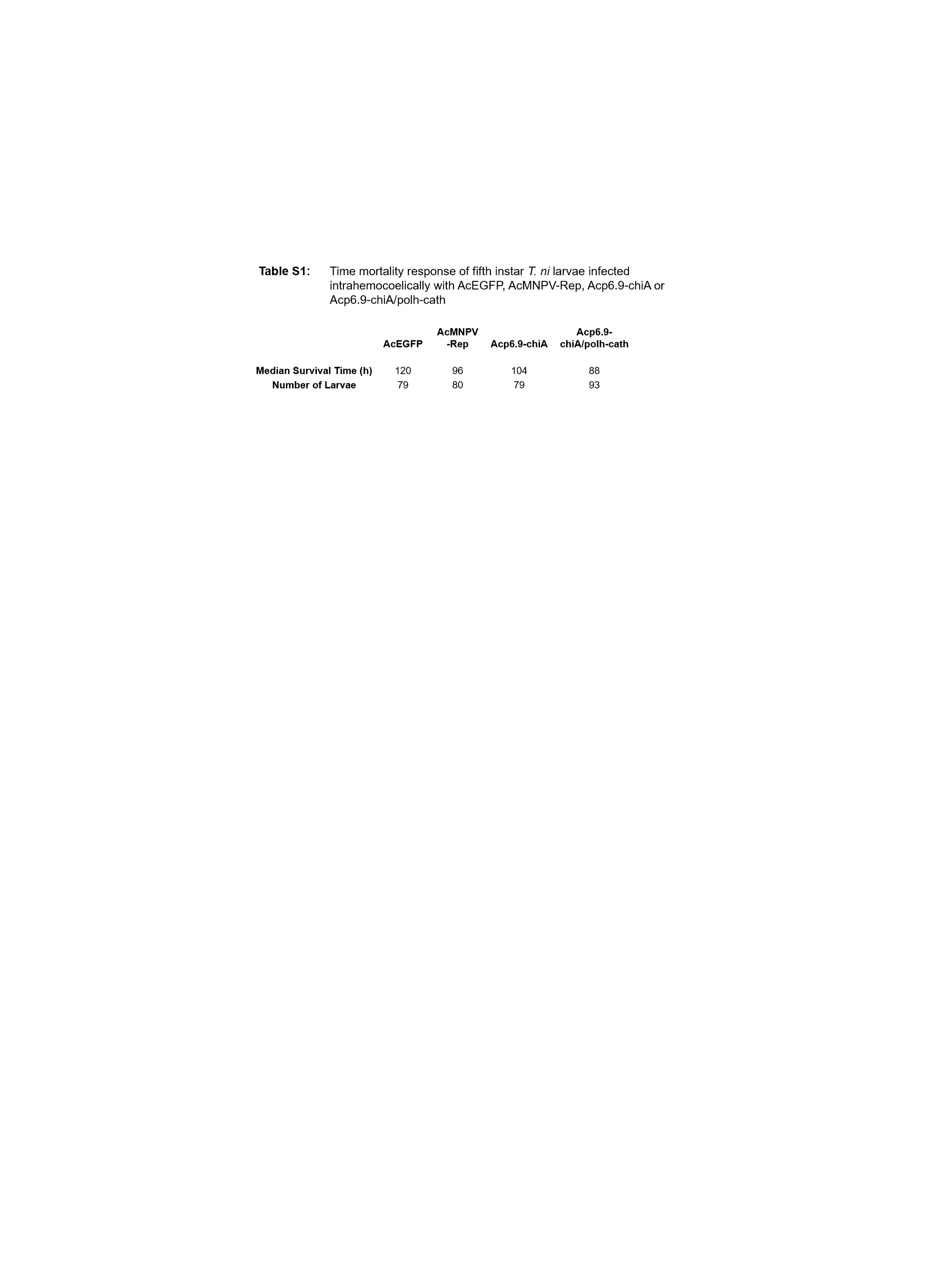

Supplement: Supplementary file 1 [file viruses-15-00503-s001.zip › viruses-2139603-supplementary.pptx]
